# Indicators of an Integrated Home Care Model Shaped by the Needs of Patients Discharged from the Emergency Department

**DOI:** 10.5334/ijic.5480

**Published:** 2020-11-25

**Authors:** Katarzyna Szwamel, Donata Kurpas

**Affiliations:** 1 Institute of Health Sciences, Univeristy of Opole, Opole, PL; 2 Department of Family Medicine, Wroclaw Medical University, Wroclaw, PL

**Keywords:** delivery of health care, integrated care, emergency service, social environment, social support, socioeconomic factors

## Abstract

**Introduction:**

Developing community care models aims to satisfy the needs of patients’ in-home care comprehensively. This is crucial to decrease adverse events and prevent rehospitalization.

**Methods:**

A cross-sectional study was conducted among 200 emergency department patients (EDPs) and 200 general practice patients (GPPs). The modified version of the Camberwell Assessment of Need Short Appraisal Schedule (CANSAS), the Health Behavior Inventory (HBI), the Generalized Self-Efficacy Scale (GSES), the Patient Satisfaction Questionnaire (PSQ), and the Multidimensional Health Locus of Control Scale (MHLCS) were used.

**Results:**

The study indicated the higher level of unmet needs in EDPs than in the population of GPPs (p = 0.008). The unmet needs increased risk of hospitalization in both groups: OR = 0.28 [95%CI 0.15–0.52] for EDP and OR = 0.33, [95%CI 0.17–0.62] for GPPs groups. We also found a significant relationship between the low levels of needs satisfaction and social-demographic variables, including health profile and the level of health behavior, generalized self-efficacy, health locus of controls, and healthcare measures in general practice.

**Discussion and Conclusion:**

We suggest that the identified factors should be included into the integrated community care model to advance satisfaction of patients’ needs, especially in patients discharged from an emergency department.

## Introduction

### Background

The growing burden of chronic diseases, patients experiencing fragmented care, and increasing demand for coordination across providers in the health and social sector correlates with the need for the integration of care. The starting point in developing an integrated care strategy should be identifying and assessing population needs [1].

Models of integrated care may enhance patient satisfaction, increase the perceived quality of care, and enable access to services [2]. The term ‘new models of care’ refers to a wide range of interventions aiming to address issues of integration across healthcare and between health and social care. Improved discharge planning and flow of care, and improved sharing of knowledge between practitioners, are essential components of new models of integrated care [3]. Discharge of the patient from the hospital to the community is critical in patient care, especially for patients with multiple comorbidities, elderly patients, or those with impaired function. Inappropriate discharge destination and incomplete communication with patients and ambulatory care can lead to adverse outcomes (e.g., emergency department [ED] visits and adverse events) [4]. Previous studies have shown that the rate of adverse events among home care patients is between 10% and 13%. The most common adverse events were falls, wound infections, psychosocial, behavioral or mental health problems, and adverse outcomes from medication errors. Between 32% and 56% of adverse events are preventable [5]. Achieving this goal is possible by ensuring safe, high-quality healthcare at the patient’s home. Home care decreases costs improves health outcomes and is connected with high levels of patient satisfaction [6]. Hence, the patient should receive in-home healthcare with simultaneous activities focused on the needs of patients and their families.

The notion of ‘*need*’ in healthcare is defined as the capacity to benefit. If health needs are to be identified, an effective intervention should be available to meet these needs and improve health [7]. Unmet needs are defined as “the difference between services judged necessary to deal appropriately with health problems and services actually received” [8]. Herr et al. (2014) claim that unmet healthcare needs are situations in which a participant needed healthcare but did not receive it [9]. An *unmet* need, as opposed to a *met* need, indicates a serious problem that was not effectively targeted in treatment [10]. Unmet needs may worsen the patient’s quality of life [11], increase the risk of hospital admissions and readmissions [12], and increase the risk of mortality [13]. Unmet needs are also an independent predictor of ED visits [14]. An especially high level of needs may be observed in people who are 65 or older and have a least 3 chronic diseases *(high-need patients)*. The Commonwealth Fund Survey shows that one-fifth of patients with a high level of needs report to the ED with health problems that could be treated at the outpatient level. This is likely to be the result of experiencing fragmentary care [15]. The coordination of care of “*high-need”* patients, however, requires an objective and complex evaluation of needs. Such evaluation allows identification of patients and groups requiring actions targeted at prevention and early intervention, quantifying the unmet needs that enable adequate allocation of resources, and a direct indication of the necessary resources [16]. In coordinated healthcare systems, the evaluation of all the social, psychological, and healthcare needs and the needs concerning the organization of life falls within the responsibilities of the care coordinator [17,18].

Previous studies have shown that satisfying patients’ needs can result from both the healthcare system (accessibility, acceptability, and availability) and individual approaches and characteristics of patients [19]. The factors determining the level of needs of patients include functional restrictions, the occurrence of mental illnesses (e.g., anxiety, depression, and bipolar disorder), multimorbidity, sociodemographic factors (patient’s age, gender, income, and education), indicators of the quality of care (i.e., relationships with the general practitioner [GP], availability of general and specialist care, the complexity of care, and support from a care coordinator) as well as the type of insurance [20,21].

### Problem Statement

The development of community care model that aims to comprehensively satisfy the needs of patients’ in-home care is crucial to decrease adverse events and prevent rehospitalization, including at the ED. It should describe integrated actions within the scope of health and social care to those patients based on the appropriate care measures. Therefore, we suggest the development of a holistic model of care which would specify the level of needs of patients in connection to their sociodemographic profile, level of health behavior, perception of the quality of the services offered by the general care, as well as patients’ individual convictions and expectations of health and social care. Note that ED in Poland is a part of the State Medical Rescue System (SMRS), which includes hospital emergency departments, medical emergency teams, including airborne medical emergency teams. The SMRS system can cooperate with trauma centers and hospital departments to treat sudden health and life-threatening conditions.

Since 1991 Poland has experienced a progressive disintegration of the social care and healthcare systems, being supervised by the government with two separate ministries. This policy has led to the loss of the connection between the physician and the division of social care workers [22]. Thus, the development of effective community care models and identification of their relevant socio-clinical factors is especially crucial for Polish patients.

Undertaking an integrated health and social care policy should be based on objective data. The study assumed that the identification of needs requiring the satisfaction of a patient discharged from ED, quantification of the level of dissatisfaction in particular areas with the use of standardized tools, and identifying factors determining their satisfaction would allow developing a model of community care. The application of such a model in-home care will allow for personalized adjustment of health and social services to a given patient and more effective use of limited material, human and financial resources of both sectors mentioned above, aimed at a given need.

The research aimed to determine and compare: (1) the level of unmet needs in a group of ED patients and a group of general practice patients; (2) factors determining the level of unmet needs as elements of an integrated model of community care for a patient discharged from the ED; and (3) the chances of hospitalization in both groups depending on the level of satisfaction of the need.

## Research Methods

### Study Design

Cross-sectional, observational studies were carried out among the inhabitants of the Kędzierzyńsko-Kozielski district (Opolskie Voivodship, Poland).

### Setting

The research was carried out between 2014 and 2016 after obtaining the consent of the Bioethics Committee at the Wroclaw Medical University (approval no. KB-87/2016) while maintaining the requirements of the Declaration of Helsinki of 1975 (amended in 2000) and Good Clinical Practice. Two groups of patients were examined. Both groups were chosen from the same population and monitored simultaneously (parallel groups). One group consisted of patients from the Emergency Department of the hospital in Kędzierzyn-Koźle (the ED group). The other group consisted in patients from four different general practice clinics, including two clinics in cities and two in villages (the GP group) in the Kędzierzyńsko-Kozielski district (Opolskie Voivodship, Poland). The method of random selection was applied to both groups of patients.

### Participants

The patients included in the study were over 18 years old, verbally responsive, provided informed consent to participate in the study, and were native speakers of Polish. Being an ED patient in the case of the study group and being a general practice patient in the control group was the basic criterion of inclusion in each group. The following people were excluded from the study: people under 18 years old, those without logical contact, people who were not users of the Polish language, and people who did not consent to take part in the study, as well as patients with difficulties which made participation in the study impossible (e.g., visual disorders reported by the patient, foreign objects in the eye, severe trauma, or patients in a critical condition). Participation in the study was offered only to those patients who fulfilled the criteria for inclusion in the study.

### Study Size

In the early stages, an invitation to participate in the study was accepted by 445 randomly chosen ED patients and 280 randomly chosen general practice patients. The study was carried out by nurses employed in the studied clinics. They were previously trained in the way the study was carried out.

### Variables

The following categories of variables were distinguished:

- The level of patients’ needs: Camberwell Index (CI).
- Sociodemographic variables (age, gender, the financial status of the family, the number of people living in one household, place of residence, marital status, relationship status, and the distance from the place of residence to the general practice and ED).
- Variables determining the health profile and the level of health behavior of the study participants i.e. treatment of chronic diseases, the number of medicines taken continually per day, treatment in a specialist clinic, the number of all hospitalizations within the last 3 years, the number of hospitalizations at an ED within the last 3 years, body mass index (BMI), systolic blood pressure, diastolic blood pressure, results of laboratory tests (International Normalized Ratio [INR]: creatinine, serum glucose concentration), general indicator of health behavior (HB), an indicator of healthy eating habits (HEH), an indicator of preventive behavior (PB), an indicator of positive psychical mental attitude (PMA), and an indicator of health practices (HP).
- Variables connected with the quality of healthcare provided by the personnel of general practice: the level of satisfaction of care provided by the general practice personnel, execution of home care by the GP, health education provided by the general practice team, and perception of the GP as a continuator of treatment (continuity of care).
- Variables connected with beliefs and expectations of patients: General Self-Efficacy Scale (GSES), the positioning of health surveillance in the dimension “internal control” (MHLC-W), the positioning of health surveillance in the dimension “the influence of others” (MHLC-I), and the positioning of health surveillance in the dimension “case” (MHLC-P).

### Data Sources

The Camberwell Assessment of Need Short Appraisal Schedule (CANSAS), the Health Behavior Inventory (HBI), the General Self-Efficacy Scale (GSES), the Patient Satisfaction Questionnaire (PSQ), and the Multidimensional Health Locus of Control Scale (MHLCS) and the questionnaire developed by the authors for sociodemographic and clinical data were used.

INR values were obtained using Dade Innovin Reagent. The proceedings were carried out according to the World Health Organization and the International Committee of Thrombosis and Hemostasis. Levels of blood serum creatinine were obtained by means of an enzymatic test on the Beckman Coulter AU machine. Glucose marking was carried out by means of the colorimetric method with glucose oxidase and the application of the Liquick Cor-GLUCOSE diagnostic set. Blood pressure was determined by means of a clock blood-pressure monitor TM-Z made by TechMed. Height and weight were measured using column scales (Seca 711) and a measuring rod (Seca 220; EN 4551).

The CANSAS was applied to evaluate the needs of ED and general practice patients (Cronbach’s alpha: 0.82). The modification of CAN (Camberwell Assessment of Needs) is focused on 22 problem fields. It evaluates the medical, psychological, environmental, and social needs of the patient. In this research, the Camberwell Index was calculated. The calculations consisted of the determination of the number (N) of met (1) and unmet (0) needs of the patient on the basis of 24 questions identifying 22 needs. Consecutively, within the (N) of needs indicated by the studied person, the number (M) of met needs (1) was established. The M/N formula was used to calculate the Camberwell Index. According to the Camberwell Index, lower average values indicate a low level of met needs, whereas higher average values indicate a high level of met needs [23].

The HBI consists of 24 statements and measures four categories of health behavior: healthy eating habits (HEH), positive mental attitude (PMA), preventive behavior (PB), and health practices (HP). Patients were asked to evaluate the frequency of activities connected with health according to a five-grade scale in which “1” meant “hardly ever,” 2 “rarely,” 3 “from time to time,” 4 “often” and 5 “almost always.” The value of the general indicator of health behavior is within the range of 24–120 points. The higher the value of the indicator, the better the health behavior. Cronbach’s alpha is 0.85 [24].

The PSQ focuses on the care provided by general practice personnel was created based on the EUROPEP questionnaire [25], the Medical Interview Satisfaction Scale [26], the Quality Assurance Program Questionnaire of the Columbia Medical Plan, U.S. [27], and the Questionnaire for Patients’ of the American Society of Internal Medicine, Family Practice Clinic – patient satisfaction questionnaire from the University of Oregon [28]. The internal cohesion (Cronbach’s alpha) is 0.94. The questionnaire consists of modules concerning patient’s subjective and objective impressions during the appointment, the cooperation of the physician with the patient during the diagnostic procedures and treatment, psychosocial factors (e.g., interest in the patient’s material and personal situation, providing information about cheaper medication), the possibility to obtain the physician’s help in urgent situations, and contact with other members of staff of the general practice [29,30]. Patients received 2 points if they answered “yes,” 1 point if they answered “sometimes,” and 0 points if they answered “no.” The study participant could obtain a score from 0 to 72 points, with higher scores indicating a higher level of patient satisfaction. Two levels of satisfaction were assumed: high (for values above the median) and low (for values equal to or lower than the median).

The GSES measures the strength of the individual’s general conviction about the effectiveness of dealing with difficult situations and obstacles. The task of the study participant is to choose the answer by circling it according to a scale in which 1 means “no,” 2 “probably not,” 3 “probably yes,” and 4 “yes.” The sum of the responses provides the general self-efficacy indicator. The result falls within 10 and 40 points. The more points, the higher the feeling of self-efficacy. Internal cohesion measured with Cronbach’s alpha is 0.85 [24].

The MHLCS consists of 18 statements and covers convictions concerning the expectations in 3 dimensions of health surveillance positioning: Internal (I) – control over one’s own health depends on the study participant; the influence of others (O) – one’s own health is the result of the influence of others, especially the medical personnel; accident (A) – one’s own state of health is governed by accident. Every sub-scale consists of six statements. The study participant rated each statement from 1 to 6 points, in which 1 means “I absolutely disagree,” 2 “I disagree to some extent,” 3 “I disagree to a small extent,” 4 “I agree to a small extent,” 5 “I agree to some extent,” and 6 “I absolutely agree.” The range of results is from 6 to 36 points. The higher the result, the stronger the conviction about the influence of one factor on their state of health.

### Statistical Analysis

The measure of average distribution was calculated for quantitative variables, while for qualitative variables, cardinality and interest were determined. In this study, qualitative variables were all observable qualities and characteristics of a sample population: gender, place of residence, marital status, relationship status, education. Qualitative data were presented in the form of the numbers (n) and percentages (%). The normality of the distribution of quantitative variables was determined using the Shapiro-Wilk test.

The Chi-square test was applied to verify the probability and differences between the structure indicators in both groups and to verify the similarities and differences between the groups for categorical features when the number of categories was larger than 3. When the number of categories was less than or equal to 3, Fisher’s exact test of independence was used.

Wilcoxon’s many-one rank test was used to test the relevance of differences between the median values of Camberwell Indexes for the ED and GP groups.

The Rho Spearman’s ratio was applied to determine the study strength and direction of correlation in variables influencing the level of meeting patient needs. The strength of the dependence was interpreted according to the following scheme: |r| ≥ 0.9 – very strong dependence, 0.7 ≤ |r| < 0.9 – strong dependence, 0.5 ≤ |r| < 0.7 – average dependence, 0.3 ≤ |r| < 0.5 – weak dependence, |r| < 0.3 – very weak dependence [30].

Correspondence analysis was used to establish the variables which appear most frequently with high and low levels of meeting patients’ needs. Only those variables which correlated on a statistically significant level with the given category were chosen for the correspondence analysis. The values were deemed high if they were higher than the median, whereas values lower than or equal to the median were considered low.

Logistic regression was used to investigate the probability of hospitalization. The median Camberwell Index values were employed to calculate the hospitalization chances of ED and GP groups.

## Results

### Participants

**Figure 1:**
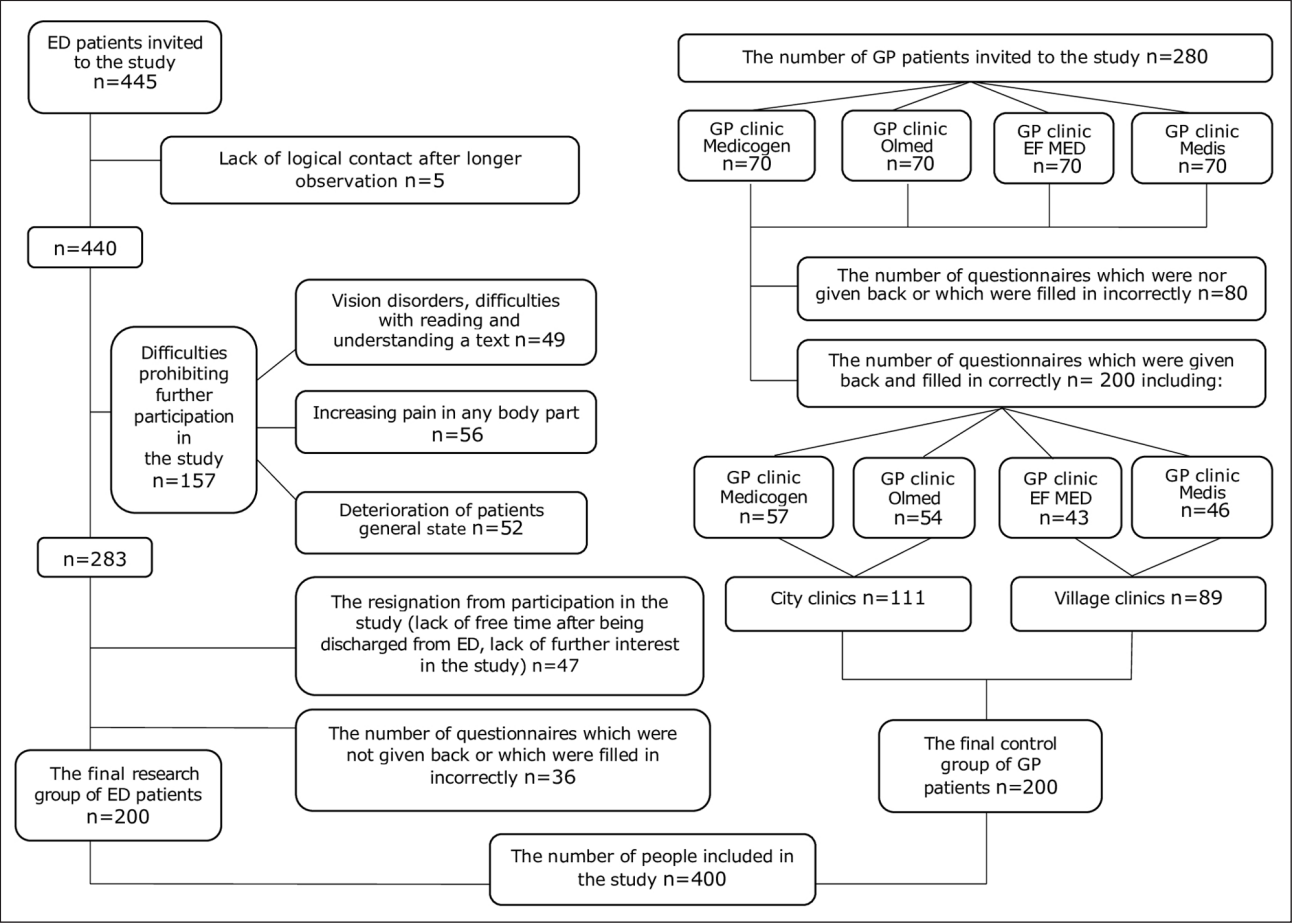
The scheme of the selection of study participants in ED and GP groups.

Out of 445 patients, 200 were eligible to be included in the ED group. In the GP group, out of 280 distributed surveys, only 200 were correctly filled in. The reasons for excluding patients from the study at each stage are represented in Figure 1.

### Descriptive Data

There were no significant differences between the ED and GP groups in terms of age, the number of people living in one household, the distance from the place of residence to the general practice, and the distance from the place of residence to the ED. The groups did not differ in their gender, education, marital status, the financial status of the family, and life in a stable relationship. There was a difference between the ED and GP groups for the size of the place of residence (*p* = 0.031) (Table 1).

**Table 1:** Structure indicators in ED and GP groups according to one feature of discrete variables.

Feature		GP		ED		Total		Test

		n	%	n	%	n	%	

Gender	Female	117	58.5	99	49.5	216	54.0	Chi^2^ = 2.91 df = 1 p = 0.087
	Male	83	41.5	101	50.5	184	46.0	
	**Total**	200	100.0	200	100.0	400	100.0	
Education	Primary	14	7.1	17	8.7	31	7.9	Chi^2^ = 1.85 df = 3 p = 0.605
	Vocational	47	24.0	53	27.0	100	25.5	
	Secondary	88	44.9	89	45.4	177	45.2	
	Higher	47	24.0	37	18.9	84	21.4	
	**Total**	**196**	**100.0**	**196**	**100.0**	**392**	**100.0**	
Marital status	Unmarried	47	23.7	41	20.8	88	22.3	Chi^2^ = 6.95 df = 3 p = 0.074
	Married	116	58.6	116	58.9	232	58.7	
	Divorced	7	3.5	19	9.6	26	6.6	
	Widowed	28	14.1	21	10.7	49	12.4	
	**Total**	**198**	**100.0**	**197**	**100.0**	**395**	**100.0**	
Financial status	Very bad	2	1.0	0	0.0	2	0.5	Chi^2^ = 7.83 df = 4 p = 0.098
	Bad	9	4.6	14	7.2	23	5.9	
	Average	84	42.6	102	52.3	186	47.4	
	Good	93	47.2	71	36.4	164	41.8	
	Very good	9	4.6	8	4.1	17	4.3	
	**Total**	**197**	**100.0**	**195**	**100.0**	**392**	**100.0**	
In a relationship	Yes	129	65.2	140	72.5	269	68.8	Chi^2^ = 2.15 df = 1 p = 0.142
	No	69	34.8	53	27.5	122	31.2	
	**Total**	**198**	**100.0**	**193**	**100.0**	**391**	**100.0**	
Place of residence	>100 000 inhabitants (city)	12	6.0	15	7.5	27	6.8	Chi^2^ = 8.86 df = 3 p = 0.031
	20 000–100 000 (medium town)	84	42.0	99	49.7	183	45.9	
	Less than 20 000 (small town)	15	7.5	24	12.1	39	9.8	
	Village	89	44.5	61	30.7	150	37.6	
	**Total**	**200**	**100.0**	**199**	**100.0**	**399**	**100.0**	

**Key:** n – cardinality, Chi^2^ – chi-square test, p – significance level, df – the number of degrees of freedom.

## Main Results

### The Level of Unmet Needs

ED patients showed a lower level of needs satisfaction than the GP group (the mean Camberwell Indices were M = 0.75 and M = 0.80, p = 0.008) (Table 2).

**Table 2:** The differences in ED and GP groups regarding the level of meeting needs.

Variable	group	n	M	SD	Me	min	max	test W p	test SW p

Camberwell Index of Needs	GP	200	0.80	0.15	0.83	0.33	1.00	**0.008**	**<0.001**
	ED	200	0.75	0.19	0.80	0.21	1.00		**<0.001**

***Key:** M – average, Me – median, SD – standard deviation, min – minimum, max – maximum, W test: Wilcoxon’s many-one rank test for the difference between medians, SW test – Shapiro-Wilk test, p – the calculated level of test significance, GP – general practice group, ED – emergency department group*.

In the ED group, unmet needs were observed in the following areas: “Having children” – 69.23% (27/39) of patients had no children but would like to have them; “medication” – 62.94% (124/197) used prescribed medication; and “psychological stress” – 57.81% (111/192) experienced psychological stress (Table 3).

**Table 3:** Met/unmet needs of ED patients.

No	Needs	Unmet (%)	Met (%)	Total (%)

1	Accommodation	13 (6.50)	187 (93.50)	200 (100)
2	Food and grocery (shopping)	34 (17.00)	166 (83.00)	200 (100)
3	Looking after the home	30 (15.00)	170 (85.00)	200 (100)
4	Self-care at home	33 (18.97)	141 (81.03)	174 (100)
4	Self-care at home	33 (18.97)	141 (81.03)	174 (100)
5	Daytime activities	72 (36.36)	126 (63.64)	198 (100)
6	Physical health	74 (37.56)	123 (62.44)	197 (100)
7	Mental health	45 (33.83)	88 (66.17)	133 (100)
8	Information on condition and treatment	18 (10.71)	150 (89.29)	168 (100)
9	Psychological distress	111 (57.81)	81 (42.19)	192 (100)
10	Drinking alcohol and problems associated with drinking	17 (14.17)	103 (85.83)	120 (100)
11	Narcotics	7 (3.7%)	183 (96.3)	190 (100)
12	Medicines that aren’t prescribed	124 (62.94)	73 (37.06)	197 (100)
13	Social life	38 (19.79)	154 (80.21)	192 (100)
14	Intimate relationships	49 (26.06)	139 (73.94)	188 (100)
15	Satisfaction with intimate relationships	36 (27.69)	94 (72.31)	130 (100)
16	Satisfaction with sexual life	65 (35.71)	117 (64.29)	182 (100)
17	Need of having children	27 (69.23)	12 (30.77)	39 (100)
18	Satisfaction with relationship with children	12 (9.38)	116 (90.63)	128 (100)
19	Possibility of communication by phone	9 (4.59)	187 (95.41)	196 (100)
20	Possibility of using public transport	40 (20.73)	153 (79.27)	193 (100)
21	Ability of budgeting own money	33 (17.19)	159 (82.81)	192 (100)
22	Getting all the money entitled to	25 (12.69)	172 (87.31)	197 (100)

For the GP patients, the strongest positive correlation with the level of needs satisfaction was observed with the following variables: positive mental attitude, the general sum of increase of health behavior, general self-efficacy, healthy eating habits, general indicator of patient’s satisfaction with services, preventive behavior, the financial status of the family (in people with very good, good and average financial status a high level of needs satisfaction was observed in comparison with people with bad and very bad financial status), and education (in people with secondary and higher education a higher level of needs satisfaction was observed than in people with primary or vocational education) (Table 4).

**Table 4:** The Spearman’s rank correlation ratio (r) for the dependence of the level of needs (Camberwell) from other variables in ED and GP patients group.

Camberwell Index (CI) and variables listed below	Name of variable	GP		ED		GP	ED	r_s_1 ≠ r_s_2?	Analysis of correspondence

		r_s_1	p1	r_s_2	p2	n1	n2	p3	

**Sociodemographic variables**									
Age	AGE	**–0.17**	0.015	**–0.32**	<0.001	200	199	0.113	Yes
Gender	GEN	**–0.16**	0.021	–0.10	0.148	200	200	0.545	Yes
Education	EDU	**0.27**	0.000	**0.35**	<0.001	196	196	0.384	Yes
Marital status	MAR	–0.06	0.407	**–0.19**	0.008	198	197	0.192	Yes
The number of people living in one household	NPH	**0.24**	0.001	**0.22**	0.002	199	198	0.835	Yes
The financial status of the family	FSF	**0.30**	<0.001	**0.35**	<0.001	197	195	0.583	Yes
Life in a stable relationship	LSR	**–0.25**	<0.001	**–0.37**	<0.001	198	193	0.192	Yes
Place of residence	POR	–0.02	0.800	–0.06	0.427	200	199	0.691	
Material status of families	MAT	**0,30**	<0.001	**0.35**	<0.001	197	195	0.583	Yes
**Variables determining the health profile and the level of health behavior**									
Treatment of chronic diseases	CHD	0.10	0.150	**0.40**	<0.001	200	200	**0.001**	Yes
The number of medication taken regularly per day	NMPD	**–0.38**	<0.001	**–0.41**	<0.001	111	96	0.802	Yes
Treatment in a specialist clinic	TSC	**0.18**	0.012	**0.38**	<0.001	199	199	**0.031**	Yes
The number of chronic diseases	NCD	**–0.23**	0.001	**–0.46**	<0.001	200	200	**0.009**	Yes
The number of all hospitalizations within last 3 years	NAH	**–0.24**	0.001	**–0.39**	<0.001	200	200	0.097	Yes
The number of ED visits within the last 3 years	NEDV	**–0.26**	0.014	**–0.31**	<0.001	86	200	0.677	Yes
Systolic blood pressure – mmHg:	SBP	–0.07	0.319	**–0.18**	0.011	199	195	0.271	Yes
Diastolic blood pressure – mmHg:	DBP	–0.11	0.124	–0.11	0.119	199	195	1.000	
INR	INR	–0.17	0.330	**–0.41**	0.001	36	67	0.218	Yes
Serum glucose concentration	SGC	–0.16	0.057	**–0.26**	0.009	136	99	0.434	Yes
Creatinine	CRE	0.01	0.952	**–0.27**	0.002	102	132	**0.032**	Yes
BMI	BMI	–0.12	0.092	**–0.27**	<0.001	198	198	0.123	Yes
IZZ – the increase of health behavior (sum of points)	IZZ	**0.49**	<0.001	**0.17**	0.020	187	179	**0.001**	
HEH – healthy eating habits	HEH	**0.39**	<0.001	0.10	0.175	198	199	**0.002**	Yes
PB – preventive behavior	PB	**0.33**	<0.001	0.10	0.168	194	190	**0.018**	Yes
PMA – positive mental attitude	PMA	**0.53**	<0.001	**0.30**	<0.001	199	195	**0.006**	Yes
HP – health practices	HP	**0.28**	<0.001	0.01	0.847	192	187	**0.007**	Yes
**Variables concerning the healthcare provided by the general practice personnel**									
The level of patients’ satisfaction with the case provided by the general practice personnel	SAT	**0.38**	<0.001	**0.14**	0.047	200	200	**0.010**	Yes
Services of the general practice team – realization of house calls	RHC	**0.20**	0.005	**0.14**	0.042	200	200	0.540	Yes
Services of the general practice team – providing health education	PHE	**0.22**	0.002	**0.18**	0.011	200	200	0.679	Yes
Services of the general practice team – perceiving the GP as a treatment continuator (continuity of treatment)	GPC	**0.16**	0.025	**0.33**	<0.001	200	200	0.072	Yes
**Variables concerning patients’ convictions and expectations**									
GSES – General self-efficacy	GSE	**0.44**	<0.001	**0.35**	<0.001	200	195	0.292	Yes
MHLC (I) dimension – internal control	MHLC-I	0.00	0.990	**0.40**	<0.001	193	196	**<0.001**	Yes
MHLC (O) dimension – influence of others	MHLC-O	–0.09	0.206	0.08	0.298	191	192	0.098	
MHLC (A) dimension – accident	MHLC-A	**–0.29**	<0.001	**–0.31**	<0.001	195	193	0.830	Yes

***Key:** GSES – the level of general self-efficacy, GP – general practice, ED – emergency department, MHLC (I) – the positioning of health surveillance in the dimension “internal control,” MHLC (O) – the positioning of health surveillance in the dimension “influence of others,” MHLC (A) – the positioning of health surveillance in the dimension “accident,” INR – international normalized ratio, n1 and n2 – cardinality of observation in the GP and ED group respectively, r_s_1 and r_s_2 – Spearman’s rank correlation ratio in GP and ED groups, p1 and p2 – the level of significance of the test verifying the null hypothesis (r = 0) in reference to the alternative that it is other than zero (r ≠ 0), p3 – the level of significance of the test verifying the null hypothesis stating that the correlation ratio r_s_1 and r_s_2 are equal (r_s_1 = r_s_2) in reference to the alternative that it is other than zero (r_s_1 ≠ r_s_2)*.

The following variables showed a significant negative correlation with the level of needs satisfaction in the GP patients: the number of medication taken regularly per day, the positioning of health surveillance in the dimension “accident,” the number of hospitalizations at the ED within the last 3 years, living in a stable relationship (people who did not live in a stable relationship more rarely obtained a high level of meeting needs than people who lived in a stable relationship), the general number of hospitalizations within the last 3 years, the number of chronic diseases, age, and gender (women obtained a high level of meeting needs more often than men) (Table 4).

For the ED patient group, the following variables correlated significantly with patient needs: the internal health locus of control, no treatment for chronic diseases, no treatment in a specialist clinic, education (people with secondary and higher education had higher levels of met needs than people with primary or vocational education), financial status (people with very good, good and average financial status were more satisfied with needs than people with bad and very bad financial status), and general feeling of self-efficacy (Table 4).

The following variables negatively correlated with the levels of met needs in the ED patient group: the number of chronic diseases, the number of medications taken regularly per day, the INR results, the general number of hospitalizations within the last 3 years, living in a stable relationship (people who did not live in a stable relationship more rarely obtained a high level of meeting needs than people who had stable relationships), age, the number of hospitalizations at the ED within the last 3 years, the health locus of control on the dimension “accident,” BMI, creatinine level, glucose concentration, marital status (a high level of meeting needs appeared less often in divorced and widowed people), and systolic blood pressure (Table 4).

### Factors Determining the Level of Needs Satisfaction: Correspondence Analysis

In both groups, the high levels of needs satisfaction (above the median) more often co-occurred with the following variables:

- sociodemographic factors: patients aged below or equal to the median (AGE–) – GP group Me = 49.00 years, min–max = 18–87 vs. ED group Me = 45.00, min–max = 18–95 years; female gender (GENf); secondary and higher education (EDU+); marital status – married (MAR+); number of people living in a household above the median (NPH+) – in the GP group Me = 3, min–max = 1–7 vs. ED group Me = 3, min–max = 1–8; good and very good material status of families (MAT+); and life in a stable relationship (LSR+).
- the health profile and level of health behaviors: lack of chronic diseases (CHD–), high values of positive mental attitude (PMA+) – GP group Me = 3.50, min–max = 1.17–5.00 vs. ED group Me = 3.50, min–max = 2.00–5.00; BMI values at a level lower or equal to the median (BMI–) – GP group Me = 25.87, min – max = 15.78–55.56 vs. ED group Me = 27.32, min – max = 16.26–66.02; the number of chronic diseases lower or equal to median (NCD–) – GP group Me = 1.00, min–max = 0.00–12.00 vs. ED group Me = 1.00, min–max = 0.00–10.00; the total number of hospitalizations over a 3-year period lower or equal to the median (NAH–) – GP group Me = 0.00, min–max = 0.00–10.00 vs. ED group Me = 1.00, min–max = 0.00–12.00; creatinine values at a level lower or equal to the median (CRE–) – GP group Me = 0.80 [mg/dl], min–max = 0.45–5.68 [mg/dl] vs. ED group Me = 0.85 [mg/dl], min–max = 0.38–4.04 [mg/dl]; systolic blood pressure lower or equal to the median (SBP–) – GP group Me = 130.00 mmHg, min–max; 90.00–190.00 mmHg vs. ED group Me = 130.00 mmHg, min–max = 90.00–230.00 mmHg; serum glucose concentration at a level lower or equal to the median (SGC–) – GP group Me = 99 [mg/dl], min–max = 50–357.70 [mg/dl] vs. ED group Me = 116.70 [mg/dl], min–max = 81.70–382.30 [mg/dl]; and high values of healthy eating habits (HEH+) – GP group Me = 3.17, min–max = 1.17 – 5.00 vs. ED group Me = 3.17, min–max = 1.17–5.00.
- quality of general healthcare measures: high level of patient satisfaction with GP services (SAT+) – in GP group Me = 52.50, min–max = 19–72 vs. ED group Me = 52.00, min–max = 18–72; GP visits at patients’ home (RHC+), providing health education by general practice doctors and nurses (PHE+), and perceiving the GP as a treatment continuator (GPC +).
- measures of patients’ beliefs and expectations: a high generalized sense of self-efficacy (GSE+) – GP group Me = 30.00, min–max = 10.00–40.00 vs. ED group Me = 31.00, min–max 13.00–40.00; a high level of locating health control in the *internal dimension* – higher than the median (MHLC-I +) – GP group Me = 25.00, min–max = 9.00–36.00 vs. ED group Me = 25.00, min–max = 9.00–36.00.

**Figure 2:**
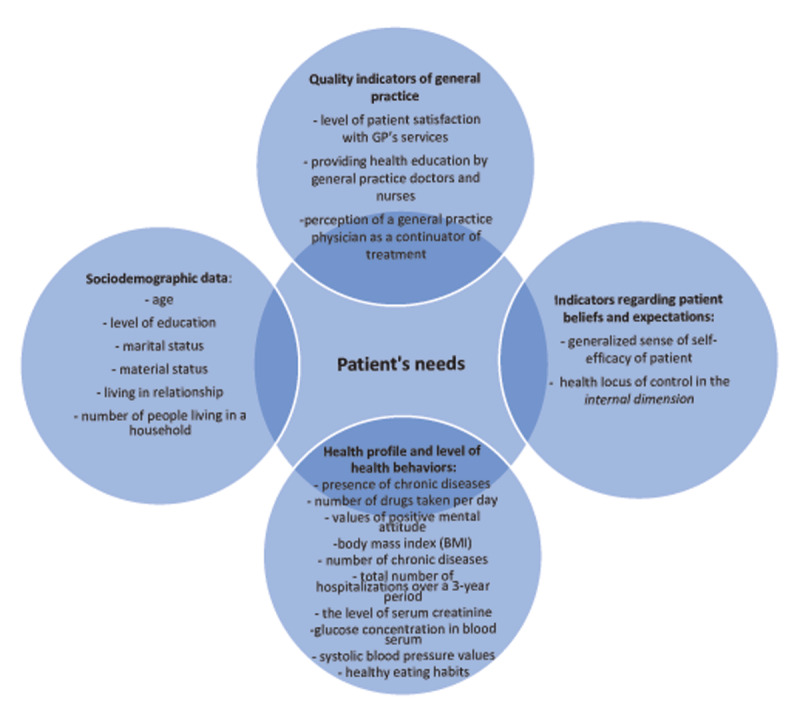
Indicators of an integrated home care model shaped by the needs of general practice patients.

The correspondence analysis in the ED group showed that for the variable “the number of medications taken regularly per day” (NMPD +, NMPD –) the unambiguous interpretation of dependence was not possible (both NMPD + and NMPD – points were positioned closer to point CI–) or the GP group, the median values of the number of used medicines was 4 compared with 5 in the ED group. The factors relevant to the integrated home care model shaped by GP patients’ needs are shown in Figure 2.

### The Chances of Hospitalization

As indicated by logical regression analysis, for GP patients with the Camberwell Index lower than 0.83, the chances of hospitalization were 3 (≈1/0.33) times higher than for patients with the index above 0.83 (OR = 0.33; 95%CI 0.17–0.62). The frequency of hospitalization in these groups was 49.0% and 24.0%, respectively (p < 0.001). Patients from the ED group with the Camberwell index lower than 0.80 had the chances of hospitalization 3.6 (≈1/0.28) times higher than patients with the index above 0.8 (OR 0.28, 95%CI 0.15–0.52). Percentages of hospitalization in these groups were 65.0% and 34.0%, respectively (p < 0.001) (Table 5).

**Table 5:** The quotient of chances of hospitalization in the ED and GP groups in relation to the level of the satisfaction of patients’ needs.

3-year hospitalization							

Variable	Me	No		Yes		OR	p

		n	%	N	%	CI1	CI2

GP group							

Camberwell Index of Needs	≤0.83	53	51	51	49	**0.33**	<0.001
	>0.83	73	76	23	24	0.17	0.62
**ED group**							

Camberwell Index of Needs	≤0.80	36	35	67	65	**0.28**	<0.001
	>0.80	64	66	33	34	0.15	0.52

***Key:** Me – median, p – the calculated level of significance of the Fisher’s accurate dependence test, OR – odds ratio, CI1 and CI2 – borders of 95% confidence interval for OR*.

## Discussion

### Key Results

The present study indicated that patients at the emergency department (ED) presented the lower satisfaction of needs than patients attending general practice (GP) (Table 2). We found that the level of needs satisfaction predicted the risk of hospitalization for both groups of patients. The important finding was that the chance of hospitalization was higher in the ED group than in the GP group (Table 5). Moreover, we have demonstrated that the level of satisfaction of patients’ needs was significantly determined by their socio-demographic factors, their health profile, their level of health behavior, general self-efficacy, health locus of control, and indicators of the quality of healthcare at the level of general practice (Table 4).

### Interpretation

The detailed analysis of patient needs at the emergency department showed dissatisfaction with social contacts (having no children and poor satisfaction with their social life and relationship with their partner) as well as dissatisfaction with physical and mental health (e.g. difficulties with using public transportation, psychological stress, lack of satisfaction with one’s health state, and using non-prescribed medication) (Table 3).

#### The Significance of Sociodemographic Indicators for the Community Care Model

The present results from either ED or GP patients identified the risk factors for unmet patient needs: adults with age over 45 (ED) or 49 (GP), male gender, primary or vocational education, small households with less than 3 people, bad financial status, and unstable relationships. The consistent correlation patterns between these risk factors and patient needs were observed in the ED and GP groups (paragraph 3.3.3).

Recent reports are consistent in distinguishing factors that positively affect the satisfaction of patient needs: high level of income, high level of education, and young age. Lower socioeconomic status generally results in higher healthcare needs and more diverse health problems [33]. Other researchers also confirmed that poor education was associated with a higher risk of having unmet needs [34]. So far, the studies reporting the effects of “gender” remain undetermined to the end. Our findings suggest that male gender and unstable relationships belong to the risk factors for poor need satisfaction. The opposite results showed that unmet needs in the Greek patient population were more frequent and significantly higher among females, married individuals, people having children, and economically inactive [24]. Similarly, a Korean study showed that women with lower income and educational levels expressed unmet healthcare needs [19]. In the same vein, studies on unmet health care needs in the Serbian population identified that the higher level of satisfaction of needs was influenced by female gender, higher education, and higher levels of material status [55]. According to this Serbian report, the least probability to report unmet healthcare needs was predicted by female gender (OR = 0.81), higher education (OR = 0.77), and the highest level of material status (OR = 0.46) [35]. Similarly, Kim et al. (2015) showed that females were more likely to experience unmet needs as compared to males [34]. In the study by Ahn et al. (2013), older women (OR = 1.831, 95%CI = 1.428–2.347) were more likely to have unmet healthcare needs than older men [36]. These gender differences may be explained at some point by a particular country or geographical region, e.g., its culture, women’s rights, views on women’s employment, etc. It should be assumed that in connection with the demographic aging of European societies, older women will experience higher levels of unmet needs than men and will require more medical, psychological, and social support [37]. It may be a result of feminization and the singularization of old age [38] as well as low self-esteemed health by women [39]. The poverty of older women is especially dangerous because they often live alone with no means to satisfy basic needs, including medication [40].

The present results in both ED and GP groups indicated that the age factor negatively correlated with the satisfaction of patient needs (Table 4). Similar findings were observed in the study of needs of patients with chronic respiratory diseases because a low Camberwell Index was more frequently reported by seniors with no relationship [31]. A previous study showed that the probability of experiencing unmet medical needs were significantly greater among older participants compared to younger [OR = 2.51, 95%CI 1.78–3.56] [32].

#### The Importance of Health Profile and Health Behavior

Our research showed that the high risk of unmet health care needs was present in populations of ED and PC patients having at least one chronic disease, hospitalized within the last 3 years in any ward, with the BMI index of overweight or obesity, diagnosed with increased creatinine level, systolic blood pressure over 130 mmHg, serum glucose concentration above acceptable parameters; individuals at risk of unmet needs had low levels of healthy eating habits and poor positive mental attitudes. All these socio-clinical factors were significantly associated with the level of needs satisfaction (paragraph 3.3.3).

Previous studies showed that unmet needs were more frequent and higher among individuals with chronic diseases [41]. The Commonwealth Fund 2014 research carried out in 11 developed countries identified socio-clinical characteristics of patients with high-level needs *(high-need patients)*. This high-need patient profile included older age over 65, diagnosis of at least 3 chronic diseases and limited self-service. High-need patients regularly used 4 or more medications, had appointments with at least 4 doctors, and were admitted to the ED multiple times within 2 years prior to/during the study. The high-need patient was also characterized by excessive usage of health benefits, frequent problems with care coordination, and financial limitations in access to care (e.g., patients did not undergo recommended tests, did not buy prescribed expensive medication, and did not attend follow-up appointments) [42].

The coexisting chronic diseases and health behavior correlated negatively with the level of satisfaction of needs also in patients suffering from respiratory system diseases. In this study, patients with a low number of chronic diseases (1) had an approximately 50-times greater chance of a high Camberwell index compared to individuals with a high number of chronic diseases (15). Individuals with a high positive mental attitude had an approximately 119-times greater chance of a high Camberwell index than individuals with low levels of these behaviors [50]. Another study on chronically ill patients in Canada showed that respondents with at least one chronic condition were more likely to report unmet needs than individuals with no chronic conditions. Moreover, chronic conditions in adults were more likely to develop unmet needs related to resource availability than those with no chronic conditions whatsoever [43].

Our results showed that overweight or obese patients were at risk of deteriorating the satisfaction of needs. High levels of unmet needs in obese patients were also reported in previous studies [44]. It is known that obesity is a causative factor in a diverse range of comorbid diseases. An overweight patient with BMI at the upper-end range may be at risk of developing metabolic syndrome, cardiovascular disease (CVD), type 2 diabetes (T2D), cancers, stroke, osteoarthritis, and respiratory problems.

Our research investigated correlations between regularly used medication and the satisfaction of patient needs. Despite the findings of the negative correlations in both groups, the correspondence analysis showed that the variable of regularly used medication was of importance for the satisfaction of needs but only in GP patients. In the GP group, usage over 4 medications corresponded with a low level of satisfaction with patient needs. However, for the ED patients, the results cannot be interpreted unambiguously. Therefore, we advise that this factor should be considered in the community care model. For example, a prospective cohort study on unmet needs for medical support in community-dwelling older adults (1,772 elderly subjects) showed that more than half of patients (1,091) experienced difficulty with self-medication. In this study, deprivation of assistance in patients who needed medical support was associated with a higher risk of hospitalization during the study period. In this study, the lack of assistance in those who needed medication assistance was associated with hospitalization during the study period [45].

#### Quality of Healthcare Provided by the General Practice Personnel

According to our study, ED and GP patients with higher risk of unmet needs were unsatisfied with the quality of benefits received in general practice; patients had no house calls from the GP (when they were necessary according to the patient’s view), had no benefits from health education, and difficulty to perceive the GP as a treatment process continuator (paragraph 3.3.3). These outcomes may result from still poor quality of primary care, reflecting the growing crisis of the healthcare system in Poland [46]. Although the organization of primary care in Poland complies with WHO general guidelines, a majority of its dimensions are negatively evaluated– the weakest part of primary care is linked with its structure, economic conditions, and coordination of care [47].

It was proved that the key features of effective general practice are to have a continuous relationship with a physician, along with first contact, comprehensiveness, and coordination of care [46]. The most important patient needs concern communication with the physician, information conveyed, and improvement in clinical outcomes [47]. It should be remembered that general practice patients come to the GP with certain expectations, and the GPs’ task should be to respond effectively to the patients’ needs [48]. The lack of responsivity of GPs regarding the patient’s expectations may turn into an increased number of ED visits. The research on the frequency of ED visits may be an example in which frequent ED visitors as likely as non-frequent visitors chose their GPs, had good access to general practices, and lasting relations with their physician, knowledgeable about their health and their personal and material situation. Although the frequent ED visitors made twice as many GP appointments, they were less willing to report that the appointment satisfied their expectations (76.12% vs. 92.53%, p < 0.001) [49]. As it can be concluded, it is not so much important the number of visits to general practice clinics as their quality. It is crucial to determine patients’ needs, especially in frequent ED visitors who claim not urgent medical issues. It is also important to provide high-quality services at the level of general practice, responding to patients’ needs and complying with current knowledge and medical art.

#### Patients’ Beliefs and Expectations

We showed that the risk group for unmet needs consists of ED and GP patients with a low feeling of self-efficacy and those who positioned the feeling of being in control in the dimension “internal” at a low level (paragraph 3.3.3). Several studies have indicated the positive associations between a high level of self-efficacy and management of chronic diseases. The previous study on patients with diagnosed diabetes showed that their maintained beliefs about own efficacy were found to be predictive for maintaining correct values of glycated hemoglobin HbA1c [50]. In another study in patients with type 2 diabetes, high general self-efficacy correlated negatively with the tendency to smoke cigarettes and positively with the tendency to do the prescribed exercises and diet. The general self-efficacy was also a significant predictor in handling asthma [51]. High levels of self-efficacy and the internal health locus of control were consistently associated with medication adherence [52]. In a group of people diagnosed with cancer, the *internal health surveillance* positively correlates with healthy eating and physical activity [53]. The studies mentioned above directly are in line with our research, indicating that patients with high self-efficacy and health locus of control are crucial components in handling one’s own health. These psychological characteristics make individuals capable of quickly and deftly react to their health needs and efficiently meet them, as well as prevent complications, especially in chronic diseases.

#### Meeting the Needs and the Number of Hospitalizations

Our results suggest that the presence of unmet needs increases the probability of patients’ hospitalization (Table 5). It was also shown that the number of annual ED admissions for older adults with unmet need for activities of daily living was higher (1.19) as compared to older adults with met needs (0.87) [14]. In another study, the unmet need for pain management was associated with more frequent ED visits [54]. Other researchers also point out the negative dependence between hospitalizations, and the satisfaction of patients’ needs, mainly at the ED [55].

### Limitations of the Study

Unmet needs are declarative and measured from the patient’s point of view in this study. Yet, a standardized measurements of patients’ needs were applied. Another limitation of the present research was the small sample of patients collected at a single ED and four general practice centers.

## Conclusion

We conclude that the effective model of community care aimed at satisfying patients’ needs discharged from the ED should embrace the relevant social-demographic characteristics: health profile and the level of health behavior, generalized self-efficacy, positioning of health surveillance/health locus of control, and measures of healthcare provided by general practitioners (GPs). The bottom line is to have these factors be included in the home care model to decrease patients’ hospitalizations. Thus, integration of actions of the ED, general practice, and community care, as well as the activity of the care coordinator, is crucial for advancing patients’ needs and decreasing the number of hospitalizations, especially at the emergency wards.

During the discharge of patients from the ED, it is advised that trained nurses should regularly screen individuals with a high level of risk of not meeting the needs in order to ensure optimal care at home. The ED screening should notably include easily and quickly determined factors at the ED conditions: the age over 45, male gender, primary or vocational education, small households, poor material status and unstable relationships, chronic illness (with at least 1 disease), hospitalization within the last 3 years, BMI indicating overweight and obesity, creatinine levels above the norm, a systolic blood pressure of 130mmHg or above, and serum glucose concentration exceeding the norm. The screening data should be relayed to the care coordinator and, if there is none, to the appropriate GP team and social welfare.

From the perspective of general practice, a care coordinator or community nurse in charge of screening should further analyze the needs of high-risk patients. Special attention should be paid to patients’ unhealthy eating habits, their poor positive mental attitudes, as well as dissatisfaction with the quality of benefits in general practice, failure to obtain house calls from the GP (in a situation when in patient’s view it is necessary), failure to obtain health education benefits, and patient’s difficulty in perceiving the GP as a treatment process continuator. The coordinator may use screening results to promptly introduce an appropriate intervention, e.g., the immediate satisfaction of formal or informal patient care needs, patient and family education, social welfare, or organizing a support group.

Personalized care adequate to the patient’s needs may result in: savings in public sector funds, higher patient satisfaction with care, achieving better health indicators by the patient, fewer hospitalizations, including rehospitalization at the ED. In the future, it is recommended to conduct the intervention studies described above on a larger scale and measure the effectiveness of interventions using objective indicators.

## Author Information

**Dr. Katarzyna Szwamel, RN, PhD** is an assistant professor at the University of Opole, Poland (Europe). Special areas of interest are: coordination of care between emergency departments and general practice, patients’ needs, health behaviors, satisfaction with care, quality of care for patients with chronic diseases, and frailty syndrome.

**Prof. Donata Kurpas, MD, PhD** is a full professor at the Wroclaw Medical University, Poland (Europe). Her interests lie in general healthcare (incl. value of care, quality of care and the indicators of healthcare effectiveness). She is interested in the public health approach within research and project design as well as the implementation of research results in community-based programs with the help of local stakeholders.
